# Supplementary material for: Association of COVID-19-Associated Pulmonary Aspergillosis with Cytomegalovirus Replication: A Case–Control Study
Source: J Fungi (Basel). 2022 Feb 6;8(2):161. doi: 10.3390/jof8020161 (PMC8877274; doi:10.3390/jof8020161)
Supplement: Supplementary file 1 [file jof-08-00161-s001.zip › jof-1555325-supplementary.pdf]

Patient	Classification	Serum GM	BAL GM	*Aspergillus* specie	Isolation sample	Antifungigram				
						Vor. MIC	Isa. MIC	Pos. MIC	Ech*. MIC	Amph.B
1	Probable	Positive	N/A	*A. fumigatus*	BAL	S	S	S	S	S
2	Probable	Negative	N/A	*A. niger*	BAL	S	S	S	S	S
3	Probable	Positive	N/A	*A. flavus*	BAL	S	S	S	S	S
4	Probable	Positive	Positive	*A. fumigatus*	BAL	S	S	S	S	S
5	Probable	Negative	N/A	*A. fumigatus*	BAL	S	S	S	S	S
6	Probable	Positive	N/A	*A. fumigatus*	BAL	S	S	S	S	S
7	Probable	Positive	N/A	*A. niger*	BAS	S	S	S	S	S
8	Probable	Negative	Positive	No culture growth		N/A	N/A	N/A	N/A	N/A
9	Probable	Negative	Positive	No culture growth		N/A	N/A	N/A	N/A	N/A
10	Probable	Negative	N/A	*A. fumigatus*	BAL	S	S	S	S	S
11	Probable	Negative	Positive	*A. niger*	BAL	S	S	S	S	S
12	Probable	Negative	N/A	*A. fumigatus*	BAL	S	S	S	S	S
13	Probable	N/A	N/A	*A. fumigatus*	BAL	S	S	S	S	S
14	Probable	Negative	Negative	*A. fumigatus*	BAL	S	S	S	S	S
15	Possible	Negative	N/A	*A. fumigatus*	Tracheal aspirate	S	S	S	S	S
16	Possible	Negative	N/A	*A. terreus*	Tracheal aspirate	S	S	S	S	R
17	Possible	Negative	N/A	No culture growth		N/A	N/A	N/A	N/A	N/A
18	Possible	Negative	N/A	*A. fumigatus*	BAS	S	S	S	S	S
19	Possible	Negative	N/A	*A. fumigatus*	Tracheal aspirate	S	S	S	S	S
20	Possible	Negative	N/A	*A. fumigatus*	BAS	S	N/A	S	S	S
21	Possible	Negative	N/A	*A. fumigatus*	Sputum	S	S	S	S	S
22	Possible	Negative	N/A	*A. fumigatus*	BAS	S	S	S	S	S
23	Possible	Negative	N/A	*A. fumigatus*	BAS	S	S	S	S	S
24	Possible	Negative	Negative	*A. fumigatus*	BAS	N/A	N/A	N/A	N/A	N/A

*Anidulafungin MIC was taken as the reference echinocandin test. If anidulafungin sensitivity was not available, caspofungin MIC was chosen.

Table S1. Patient-level data on microbiological data and available antifungigram of CAPA cases. For MIC interpretation, EUCAST 2020 criteria were used. CAPA: COVID-associated pulmonary aspergillosis; GM: galactomannan; BAL: bronchoalveolar lavage; BAS: broncoaspirate; Vor: voriconazole; Pos: posaconazole; Ech: echinocandin; AmphB: amphotericin B; MIC: minimum inhibitory concentration; N/A: not available.
